# Value of dynamic changes in inflammatory biomarkers for predicting intravenous immunoglobulin resistance in children with Kawasaki disease

**DOI:** 10.3389/fimmu.2025.1632578

**Published:** 2025-09-29

**Authors:** Zhiyuan Liu, Yidan Zhang, PanPan Liu, Weiguo Qian, Qiuqin Xu, Miao Hou, Ying Liu, Guanghui Qian, Jiajia Tan, Qianzi Ge, Mingyang Zhang, Jing Li, Sheng Zhao, Haitao Lv, Shuhui Wang

**Affiliations:** ^1^ Department of Cardiology, Children’s Hospital of Soochow University, Suzhou, Jiangsu, China; ^2^ Department of Pediatrics, Institute of Pediatric Research, Children’s Hospital of Soochow University, Suzhou, Jiangsu, China; ^3^ Department of Emergency, Children’s Hospital of Soochow University, Suzhou, Jiangsu, China; ^4^ Department of Cardiology, Anhui Provincial Children’s Hospital, Hefei, Anhui, China

**Keywords:** Kawasaki disease, fractional changes, inflammatory biomarkers, intravenous immunoglobulin, predictors

## Abstract

**Purpose:**

This study assessed the predictive value of dynamic laboratory parameter changes before and after intravenous immunoglobulin (IVIG) treatment for IVIG resistance in children with Kawasaki disease (KD).

**Methods:**

Children with KD were stratified based on the occurrence of IVIG resistance. Logistic regression analyses were conducted to identify independent risk factors. The predictive performance of variables and their fractional changes (FC) was evaluated through receiver operating characteristic (ROC) curve analysis. Nonlinear associations between predictors and outcomes were examined via restricted cubic spline (RCS) analysis.

**Results:**

The Soochow cohort analyzed 1,796 children, with IVIG resistance observed in 140 cases (7.8%). 636 children from the Anhui cohort were included in external validation. Multivariate regression analysis identified pre-treatment CLR and Hb, post-treatment CLR, LMR, NLR, Hb, and FCs in WBC, Hb, NE%, and NE count as significant independent predictors of IVIG resistance (P < 0.05). ROC analysis demonstrated that WBC(FC) and NE count(FC) were the strongest predictors of IVIG resistance, with AUCs of 0.7677 and 0.7818, respectively, outperforming other parameters. The combined AUC of FC was 0.8307 in the Soochow cohort and 0.8564 in the validation cohort. RCS analysis revealed significant nonlinear relationships between predictors and IVIG resistance.

**Conclusion:**

Fractional changes in WBC and NE count were established as robust predictors of IVIG resistance in KD. Future efforts should focus on developing predictive models with thresholds and dynamic risk assessments at various time points to enhance the accuracy of IVIG resistance prediction. Clinicians should closely monitor children with IVIG resistance risk factors and reassess the risk after first treatment.

## 1 Introduction

Kawasaki disease (KD) is a febrile illness characterized by inflammation of medium and small arteries, predominantly in children below 5 years old (1). In developed countries, KD is the primary cause of acquired heart disease in children, with coronary artery lesions (CAL) being its most serious outcome (2). In untreated cases, approximately 25% of children develop CAL, which can progress to coronary aneurysms or sudden death in severe cases (3). The etiology and precise pathogenesis of KD remain unclear. The standard treatment of high-dose intravenous immunoglobulin (IVIG) combined with oral aspirin significantly lowers the risk of CAL. Nonetheless, 10%–20% of children exhibit a lack of response to IVIG therapy, a condition known as IVIG resistance. Previous research has established that IVIG resistance markedly increases the risk of CAL development (4, 5). Consequently, early identification of IVIG resistance in children with KD is essential for timely, more aggressive intervention.

Several scoring systems have been developed in previous studies (5–8) to evaluate the risk of IVIG resistance and CAL in children with KD, incorporating various clinical features and laboratory parameters such as age, fever duration, and blood biomarkers. These systems include the Kobayashi (5), Egami (6), Formosa (7), and Sano (8) scores, as well as the San Diego scoring system (9). However, due to genetic and environmental differences, these scoring systems have demonstrated strong predictive value only in specific populations, with inconsistent performance across diverse groups, limiting their generalizability (10, 11). In addition to these established systems, certain inflammatory markers and combinations of laboratory parameters, such as the neutrophil-to-lymphocyte ratio (NLR) and platelet-to-lymphocyte ratio (PLR), have also been proposed as predictors of IVIG resistance (12, 13). A recent meta-analysis, however, revealed that the sensitivity and specificity of pre-treatment NLR and PLR in predicting IVIG resistance only were 58% and 73%, respectively, with an AUC of 0.72 (14). A multicenter study by Liu et al. suggested that the C-reactive protein (CRP) to albumin ratio (CAR) before treatment could potentially predict IVIG resistance (15). However, a systematic review reported that CAR’s sensitivity for predicting IVIG resistance was 0.65, specificity was 0.71, and the AUC was only 0.70 (16). Thus, while pre-treatment CAR may offer some predictive value, it is not yet a definitive biomarker for diagnosing IVIG resistance.

Despite significant advancements, the predictive power of these biomarkers remains suboptimal. One major limitation is that most studies have focused solely on pre-treatment laboratory parameters, failing to account for dynamic changes in these indicators. In reality, inflammatory markers fluctuate throughout the disease process, and single-point measurements may not adequately reflect the child’s inflammatory status or provide sufficient clinical insight for prediction. Laboratory parameters that vary before and after IVIG treatment may offer a more robust approach to identifying children with risk of IVIG resistance. Consequently, a large-scale observational study was conducted to assess the predictive value of laboratory parameters and their changes before and after treatment for IVIG resistance in children with KD. It is hypothesized that these laboratory parameters serve as high-risk factors for IVIG resistance and that their fluctuations before and after IVIG treatment enhance predictive accuracy for these outcomes.

## 2 Methods

### 2.1 Patients and study design

This was a two-center, retrospective cohort study conducted in China. The data from Hospital of Soochow University and Anhui Provincial Children’s Hospital were enrolled in study. In our study design, the data from Hospital of Soochow University were analyzed for risk indicators screening, and the data from Anhui Provincial Children’s Hospital were employed as validation cohort for indicators validation. The development cohort is used to explore and establish the prediction model, while the validation cohort independently tests the performance of the model to ensure the reliability and stability of the results.

Data were retrospectively analyzed from pediatric patients diagnosed with KD and hospitalized for treatment at the Children’s Hospital of Soochow University between January 1, 2020, and January 1, 2024. The validation cohort consists of children diagnosed with Kawasaki disease who were hospitalized at Anhui Provincial Children’s Hospital between December 1, 2021, and December 1, 2023. The inclusion criteria: pediatric patients diagnosed with complete or incomplete Kawasaki Disease according to the American Heart Association (AHA) criteria (17). Exclusion criteria included: (1) children who had received corticosteroids, other immunosuppressants, or blood products within one month prior to the initial IVIG treatment; (2) children who received non-standard IVIG treatment; (3) children who had received IVIG treatment prior to admission; (4) children with serious complications; (5) recurrent cases of KD; (6) children who were afebrile at enrollment or had confirmed infections; (7) children with immune deficiencies, chromosomal abnormalities, or other severe immunological disorders; (8) children discharged during the treatment or had missing data. According to the above inclusion and exclusion criteria, eligible children will be included in the final analysis. This study was conducted in accordance with the ethical standards as laid down in the Declaration of Helsinki and its later amendments or comparable ethical standards. The electronic medical records of hospital were scrutinized retrospectively to obtaining data of pediatric patients with Kawasaki. This study was approved by the Ethics Committees of the Children’s Hospital of Soochow University (approval no. 2025cs010) and the Ethics Committee of Anhui Provincial Children’s Hospital (approval no. EYLL-2021-002).The written informed consents for participation in this study was provided by the participants’ legal guardians/next of kin.

### 2.2 Definitions

IVIG resistance was defined as the persistence or recurrence of fever ≥38.0°C lasting for more than 36 hours after IVIG treatment (17). Patients with confirmed KD received a total IVIG dose of 2 g/kg and 30–50 mg/kg/day oral aspirin upon hospitalization. Aspirin dosage was tapered to 3–5 mg/kg/day once blood routine tests and CRP levels normalized, with reassessment occurring 3–4 days after fever resolution. All hospitalized patients with KD were treated following the standard specifications and guidelines established by the AHA (17). Fever resolution was defined as a temperature <37.5°C maintained for 24 hours. Coronary artery diameter measurements via echocardiography were used to calculate the Z-score using cardio Z software. CAL was defined as a Z-score ≥2 in any coronary segment, including the left main coronary artery (LMCA), left anterior descending artery (LAD), left circumflex artery (LCX), and proximal or middle segments of the right coronary artery (RCA) (18).

### 2.3 Data collection

Baseline characteristics included demographic data (age, sex, and weight), clinical manifestations at admission (e.g., cervical lymphadenitis, rash, extremity changes), and fever duration prior to admission. The diagnoses of incomplete KD or IVIG resistance were evaluated before and after the first IVIG treatment, respectively. Echocardiography was performed within two days of diagnosis and repeated prior to discharge and within four weeks after treatment. If severe complications occurred, additional echocardiograms were conducted as needed. Laboratory data included white blood cell (WBC) count, neutrophil count (NE count), lymphocyte count (LY count), platelet count, hematocrit (HCT), hemoglobin concentration, eosinophil percentage (EO%), lymphocyte percentage (LY%), neutrophil percentage (NE%), and CRP levels. The original laboratory parameters were obtained from whole blood samples (2.0ml) collected via venipuncture. According to the specifications in the manual, the parameters were measured using a fully automated hematology analyzer (Sysmex, XN-351) and a fully automated biochemical analyzer (Beckman Coulter, AU5800). In addition, some inflammatory markers were calculated: Neutrophil-to-Lymphocyte Ratio (NLR, NLR = Neutrophil count/Lymphocyte count), Platelet-to-Lymphocyte Ratio (PLR, Platelet count/Lymphocyte count), Lymphocyte-to-Monocyte Ratio (LMR, Lymphocyte count/Monocyte count), C-reactive Protein-to-Lymphocyte Ratio (CLR, C-reactive protein/Lymphocyte count), and Neutrophil-to-Platelet Ratio (NPR, Neutrophil count/Platelet count). The systemic immune inflammation index (SII) was calculated using the formula: SII = platelet count × (NE count/LY count). All laboratory data of hospitalized children were collected pre- and post-IVIG treatment, with fractional changes (FC) calculated for each variable. FC was calculated using the formula: FC = (Y–X)/X, where X and Y denote values before and within 24 hours after IVIG treatment, respectively.

### 2.4 Statistical analyses

Normally distributed data are presented as mean ± standard deviation, with group differences assessed using t-tests. Skewed data are presented as the median (interquartile range), and differences between groups were analyzed with the nonparametric rank-sum test. Categorical data are described by frequency, and differences between groups were assessed using the χ2 test or Fisher’s exact test. The variance inflation factor (VIF) was used to assess multicollinearity, and logistic regression (univariate and multivariate) analyses were performed to identify risk variables. Receiver Operating Characteristic (ROC) curves were generated to determine the optimal threshold of significant parameters, with the Area Under the Curve (AUC) calculated to evaluate the predictive capacity of risk factors. Multivariable-adjusted Restricted Cubic Spline (RCS) regression was applied to examine nonlinear associations between predictive variables and outcomes. The quantity of knots was established according to the minimal Akaike Information Criterion (AIC). Variables were regrouped according to inflection points, and outcome risk differences between subgroups were assessed.

The children in the Soochow cohort were randomly divided into a training set and an internal validation set at a ratio of 7:3. The Anhui cohort was used as the external validation set, and the variables with fractional change were selected as risk factors to establish a nomogram for predicting IVIG resistance in Kawasaki disease. The ROC curve of the model in the training set and the validation set was drawn, and the AUC was calculated to evaluate the predictive ability of the model. Bootstrap method was used to draw the correction curve of 1000 resampling times to evaluate the consistency of prediction probability and observation results, and decision curve analysis (DCA) was used to evaluate the clinical application value of the model. In addition, the nomogram also was developed as a web risk calculation tool for clinical application. A *P* value of <0.05 indicates a statistically significant difference. All analyses were performed using R (version 4.4.3).

## 3 Results

### 3.1 Baseline characteristics

**Figure 1:**
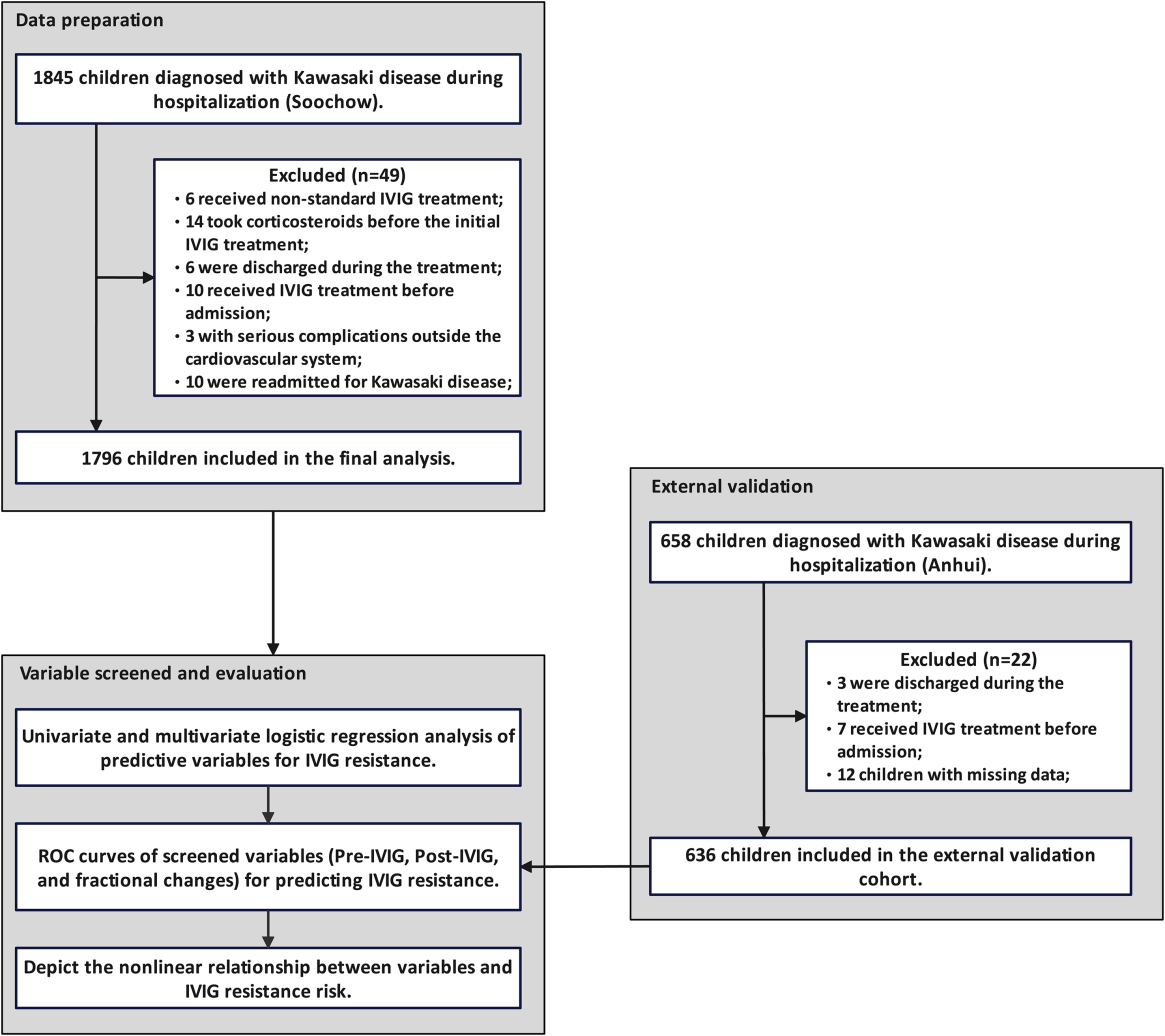
Patient selection and study flowchart. Based on the inclusion and exclusion criteria, 1,796 patients from the Soochow cohort were included in the analysis, and 636 children from the Anhui cohort were used for external validation.

Children diagnosed with KD between January 2020 and January 2024 were screened based on inclusion and exclusion criteria. A total of 1,845 children were identified, including 6 who received non-standard IVIG treatment, 14 who had prior corticosteroid use before initial IVIG administration, 6 who were discharged during treatment, 10 who received their first IVIG dose before admission, 3 with severe non-cardiovascular complications, and 10 who were readmitted for KD. None of the children were treated with infliximab, cyclosporine, anavalin, cyclophosphamide, or plasma exchange during hospitalization. Ultimately, 1,796 children were included in the final analysis, consisting of 740 females (41.2%) and 1,056 males (58.8%). The median age was 22 months (range 12–38 months), and median fever duration was 7 days (range 6–8 days) prior to admission. Among them, 140 (7.8%) presented with IVIG-resistant. In addition, among the 658 children in the external validation cohort, 3 were discharged during treatment, 7 had received IVIG treatment prior to admission, and 12 had missing data, and thus were excluded. The detailed screening process is illustrated in **Figure 1**.

Based on treatment response, children were categorized into IVIG-responsive/IVIG-resistant groups. Baseline characteristics between groups were compared (**Table 1**). Except for the presence of CAL (P = 0.004) and the duration of fever prior to admission (P < 0.05), there were no significant differences in demographic or clinical characteristics between the IVIG-responsive and IVIG-resistant groups (P > 0.05).

**Table 1:** Comparison of features and clinical manifestations between groups.

Variables	IVIG-responsive (n=1656)	IVIG-resistant (n=140)	*p*-value
Demographic characteristics			
Age in months, median (range)	22(12-38)	23.5(12-41)	0.705
Male, n (%)	972 (58.7)	84 (60.0)	0.832
Weight, kg (range)	12(10-15)	12(10-15.5)	0.189
Clinical characteristics			
Cervical lymphadenitis, n (%)	1477 (89.2)	128 (91.4)	0.495
Conjunctival injection, n (%)	1484 (89.6)	121 (86.4)	0.303
Oral mucosal changes, n (%)	1609 (97.2)	132 (94.3)	0.07
Skin rash, n (%)	1227 (74.1)	113 (80.7)	0.104
Extremity changes, n (%)	800 (48.3)	80 (57.1)	0.055
Complete KD, n (%)	1399 (84.5)	119 (85.0)	0.967
CAL, n (%)	372 (22.5)	75 (53.6)	0.004
Days of fever before admission median (range)	7 (6-8)	9 (7-10)	<0.05

Data are presented as median (range) or number (percentage).

IVIG, Intravenous immunoglobulin; CAL, Coronary artery lesions; KD, Kawasaki disease.

### 3.2 Univariate and multivariate logistic analysis of predictive variables for IVIG resistance

Compared to the IVIG-responsive group, the IVIG-resistant group showed higher levels of CRP, NE%, NLR, SII, CLR, NPR, and PLR levels, and lower levels of EO%, HCT, LY%, LY count, and platelet count before IVIG treatment(P < 0.05) (**Table 2**). Post-IVIG treatment, CRP, NE%, NLR, NPR, and PLR remained elevated in the IVIG-resistant group, while Hb, EO%, HCT, NE%, NE count, and LY% were lower compared to the IVIG-responsive group (P < 0.05). Additionally, WBC, LY count, and LMR levels were also elevated in the IVIG-resistant group (P < 0.05).

**Table 2:** Laboratory values between groups before and after IVIG.

Characteristics	Pre-IVIG		*P-* value	Post-IVIG		*P-* value
	IVIG-resistant	IVIG-responsive		IVIG-resistant	IVIG-responsive	
WBC, (x10^9/^L)	13.86(10.15, 18.6)	14.5(11.37, 18.55)	0.221	14.89 (9.43, 20.38)	8.61 (6.78,11.19)	< 0.001
CRP (mg/L)	87.77(47.42,144.13)	65.4(38.12, 101.98)	< 0.001	11.8(3.08, 26.15)	6.7(3.01, 14.29)	< 0.001
HB (g/L)	111(101, 117)	111(105, 118)	0.117	104(95, 114)	110(103, 118)	< 0.001
EO, %	0.17(0.03, 0.41)	0.2(0.07, 0.45)	0.03	0.12(0.06, 0.26)	0.25(0.14, 0.42)	< 0.001
HCT	0.33(0.3, 0.35)	0.34(0.32, 0.36)	0.028	0.32(0.29, 0.35)	0.34(0.31, 0.36)	< 0.001
NE, %	73.4(57.6, 83.75)	65.95(54.68, 76.3)	< 0.001	49.4(35.43, 59.2)	32.3(23, 44.12)	< 0.001
NE count, (x10^9^/L)	9.77(6.28, 13.03)	9.27(6.65, 12.79)	0.559	7.17(4.34, 11.22)	2.63(1.74, 4.29)	< 0.001
LY, %	18.75(10.75, 30.77)	24.7(16.4, 33.8)	< 0.001	40.05(31, 53.05)	55.3(43.9, 64.3)	< 0.001
LY count,(x10^9^/L)	2.62(1.47, 4.06)	3.45(2.22, 4.98)	< 0.001	5.58(3.9, 8.26)	4.53(3.31, 6.18)	< 0.001
Platelet count, (x10^9^/L)	295(234, 384)	338(274, 422)	< 0.001	520(406.5, 600)	517(407, 591)	0.262
NLR	3.66(1.84, 7.46)	2.66(1.6, 4.62)	< 0.001	1.23(0.66, 1.92)	0.58(0.36, 1)	< 0.001
SII	1125.78 (603.04, 2164.06)	920.74 (529.28, 1553.1)	0.011	292.66 (170.54, 501.07)	620.91 (326.15, 999.07)	< 0.001
LMR	16.68 (6.3, 61.55)	16.37 (7.81, 47.04)	0.837	47.05 (16.51, 108.35)	17.92(10.58, 32.62)	< 0.001
CLR	33.79 (13.24, 84.56)	18.57 (8.78, 37.35)	< 0.001	1.75 (0.49, 6.7)	1.52 (0.58, 3.44)	0.151
NPR	0.03 (0.02, 0.05)	0.03 (0.02, 0.04)	0.004	0.01 (0.01, 0.02)	0 (0, 0.01)	< 0.001
PLR	121.06(75.18, 195.15)	101.92 (69.44, 147.32)	0.003	106.96 (82.21, 145.74)	86.6 (65.15, 127.37)	< 0.001

IVIG, Intravenous immunoglobulin; KD, Kawasaki disease; WBC, White blood cell count; CRP, C-reactive protein; HB, Hemoglobin; EO, Eosinophil percentage; HCT, Hematocrit; NE, Neutrophil percentage; NE count, Neutrophil count; LY, Lymphocyte percentage; LY count, Lymphocyte count; NLR, Neutrophil-to-lymphocyte ratio; SII, Systemic immune-inflammation index; LMR, Lymphocyte-to-monocyte ratio; CLR, C-reactive protein-to-lymphocyte ratio; NPR, Neutrophil-to-platelet ratio; PLR, Platelet-to-lymphocyte ratio.

Univariate regression analysis identified statistically significant indicators before IVIG treatment, including CLR, PLR, PLT, LY count, LY%, NE%, Hb, and EO% (P < 0.05). Post-IVIG treatment, CLR, LMR, SII, PLR, NLR, PLT, HCT, and Hb were found to be statistically significant (P < 0.05). In addition, we also analyzed the fractional changes of variables before and after IVIG treatment. The results showed that fractional change of WBC, Hb, CRP, NE%, NE count, PLR and LMR had significant differences between the IVIG resistant group and the IVIG responsive group. These variables were subsequently included in multivariate logistic regression analysis, followed by a collinearity test for all variables. After adjusting for confounding factors such as age, sex, and body weight, multivariate analysis demonstrated that pre-treatment CLR and Hb, post-treatment CLR, LMR, NLR, and Hb, as well as fractional changes in WBC, Hb, NE%, and NE count, were significant independent predictors of IVIG resistance (P < 0.05). Collinearity analysis confirmed that the VIF for all variables was less than 5, indicating no significant collinearity among them (**Supplementary Table 1**).

### 3.3 Predictive value for IVIG resistance

**Figure 2:**
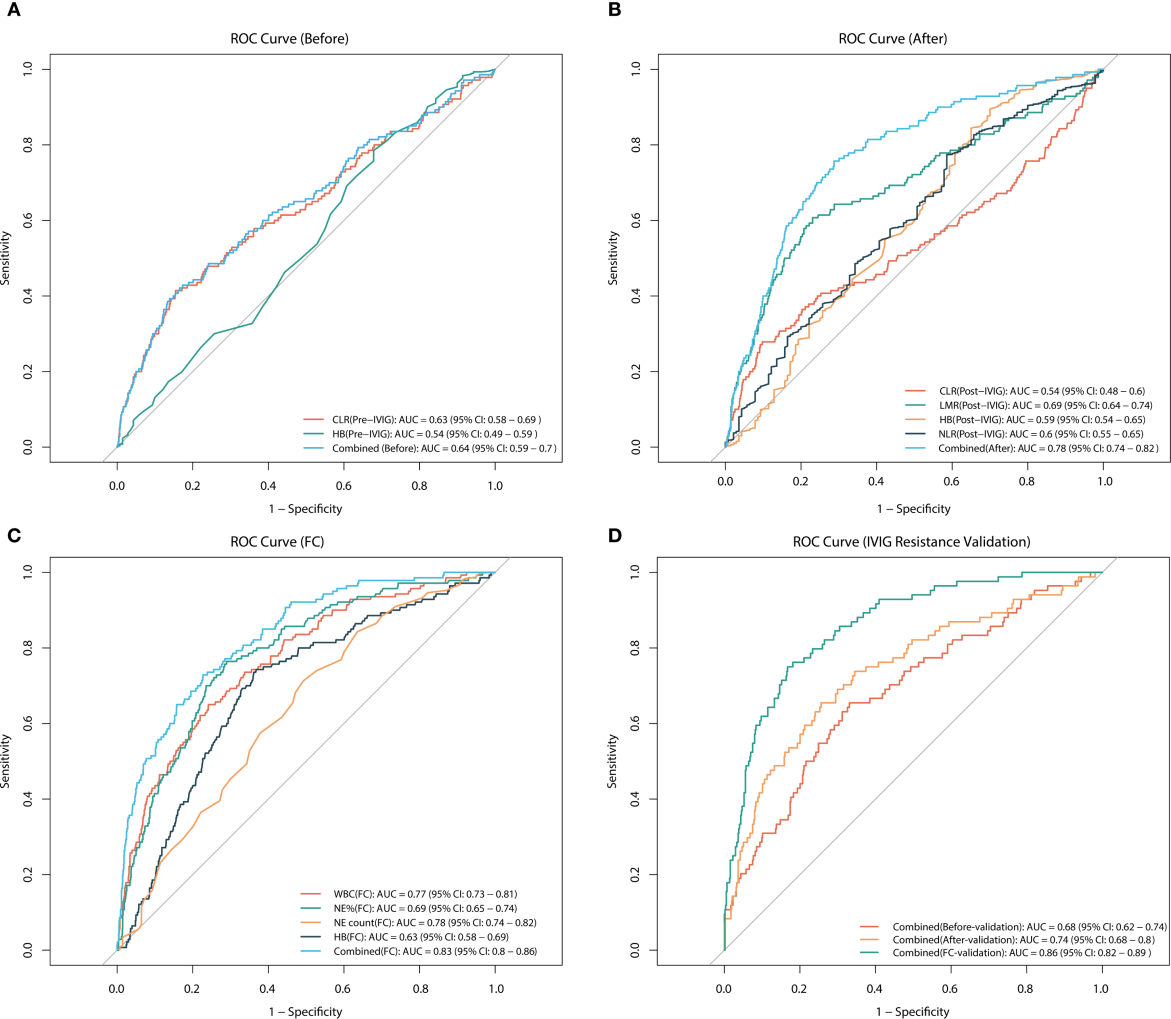
ROC curves for predicting IVIG resistance. **(A)** ROC analysis for pre-treatment variables, including CLR, Hb, and their combined, in predicting IVIG resistance. **(B)** ROC analysis for post-treatment variables, including CLR, LMR, Hb, NLR, and their combined, in predicting IVIG resistance. **(C)** ROC analysis for the fractional changes in WBC, NE%, NE count, and Hb, as well as their combined, in predicting IVIG resistance. **(D)** ROC analysis of validation cohort for predicting IVIG resistance using combined variables (before, after, and FC). CLR, C-reactive protein-to-lymphocyte ratio; NE, neutrophil; WBC, white blood cell count; PLT, platelet count; Hb, hemoglobin; LMR, lymphocyte-to-monocyte ratio; NLR, neutrophil-to-lymphocyte ratio; IVIG, intravenous immunoglobulin.

To assess the value of risk factors identified through multivariate regression analysis in predicting IVIG resistance, ROC curves were plotted for these factors before and after IVIG treatment, as well as their fractional changes (**Table 3**, **Figure 2**). **Figure 2A** presents the ROC curve for pre-IVIG risk factors predicting IVIG resistance. The optimal cutoff value for CLR before IVIG is 50.26, yielding a sensitivity of 41.43% and a specificity of 84.54% (AUC = 0.63; 95% CI, 0.581–0.688). The combined AUC of the risk factors before IVIG treatment was 0.643, with a sensitivity of 42.14% and a specificity of 83.45%. **Figure 2B** shows the ROC curve for post-IVIG risk factors predicting IVIG resistance. The optimal cutoff for LMR after IVIG is 34.789, with a sensitivity of 60.71% and a specificity of 76.93% (AUC = 0.691; 95% CI, 0.638–0.743). Similarly, the optimal cutoff for Hb after IVIG is 104.5, demonstrating a sensitivity of 89.55% and a specificity of 30% (AUC = 0.59; 95% CI, 0.583–0.685). The combined AUC of the risk factors after IVIG treatment was 0.7762, with a sensitivity of 75.71% and a specificity of 71.25%. **Figure 2C** displays the ROC curves for the FC predictive variables. WBC(FC) exhibited a threshold of -0.151, with a sensitivity of 65.00% and a specificity of 75.85% (AUC = 0.7677; 95% CI, 0.7267–0.8088), indicating strong predictive performance. NE%(FC) demonstrated a threshold of -0.419, achieving a sensitivity of 74.29% and a specificity of 63.22% (AUC = 0.6929; 95% CI, 0.6489–0.737), while NE count(FC) showed the strongest predictive capacity for IVIG resistance, with a threshold of -0.5505, sensitivity of 76.43%, and specificity of 71.01% (AUC = 0.7818; 95% CI, 0.7432–0.8204). The combined AUC of FC predictive variables was 0.8307, with a sensitivity of 72.86% and a specificity of 77.29%. In the external cohort, we validated the combined predictive ability of risk factors before and after IVIG treatment, as well as fractional changes (**Figure 2D**). For the risk factors prior to IVIG treatment, the combined AUC in the validation cohort was 0.6848, with a sensitivity of 65.48% and a specificity of 66.89%. The combined AUC of the risk factors after IVIG treatment was 0.7412, with a sensitivity of 65.48% and a specificity of 74.39%. For the FC predictive variables, the combined AUC reached 0.8564, showing a sensitivity of 75.0% and a specificity of 83.45%. Overall, the combined predictive value of the three types of variables (Pre-, Post, and FC) is generally consistent with the above results.

**Table 3:** ROC analysis of predictor variables for IVIG resistance.

Variable	Sensitivity	Specificity	AUC	95% CI Lower	95% CI Upper
Before IVIG					
CLR	0.414	0.845	0.634	0.581	0.688
HB	0.785	0.321	0.54	0.487	0.593
Combined (Before)	0.421	0.835	0.643	0.591	0.696
After IVIG					
CLR	0.279	0.902	0.537	0.477	0.596
LMR	0.607	0.769	0.691	0.638	0.743
HB	0.896	0.3	0.592	0.538	0.647
NLR	0.774	0.414	0.6	0.549	0.648
Combined (After)	0.757	0.713	0.776	0.736	0.817
Fractional change					
WBC	0.65	0.759	0.768	0.727	0.809
NE%	0.743	0.632	0.693	0.649	0.737
NE count	0.764	0.71	0.782	0.743	0.820
HB	0.713	0.507	0.634	0.583	0.685
Combined (FC)	0.729	0.773	0.831	0.800	0.864

IVIG, Intravenous immunoglobulin; HB, Hemoglobin; CLR, C-reactive protein-to-lymphocyte ratio; LMR, Lymphocyte-to-monocyte ratio; NLR, Neutrophil-to-lymphocyte ratio; WBC, White blood cell count; NE%, Neutrophil percentage; NE count, Neutrophil count; AUC, area under the curve; CI, confidence interval; FC, fractional changes.

### 3.4 The detection of nonlinear relationships

**Figure 3:**
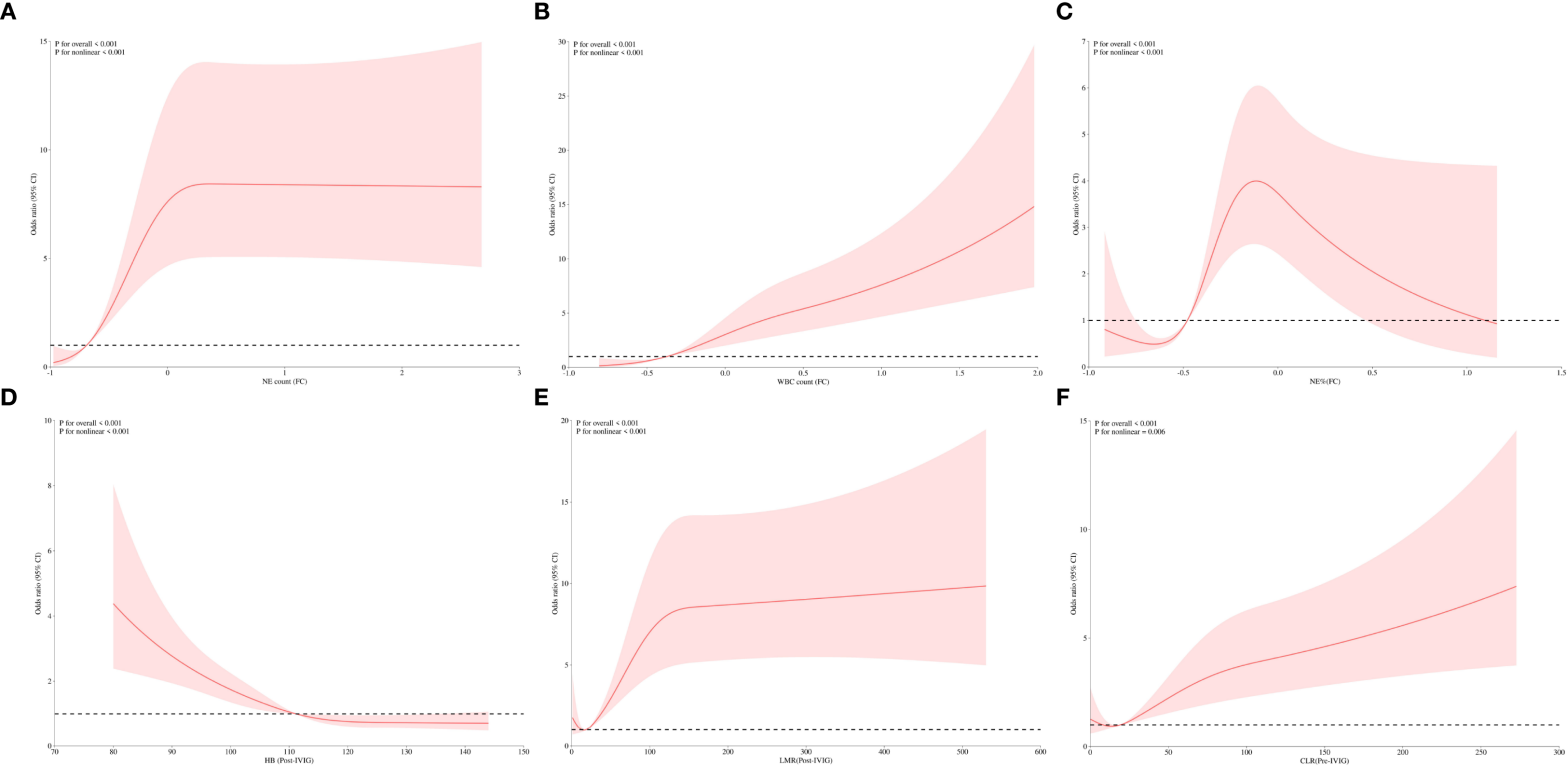
RCS analysis of predictive variables for IVIG resistance. **(A)** Correlation of NE count (FC) with IVIG resistance. **(B)** Correlation of WBC (FC) with IVIG resistance. **(C)** Correlation of NE% (FC) with IVIG resistance. **(D)** Correlation of Hb (Post-IVIG) with IVIG resistance. **(E)** Correlation of LMR (Post-IVIG) with IVIG resistance. **(F)** Correlation of CLR (Pre-IVIG) with IVIG resistance. NE count, neutrophil count; WBC, white blood cell count; NE%, neutrophil percentage; Hb, hemoglobin; LMR, lymphocyte-to-monocyte ratio; CLR, C-reactive protein-to-lymphocyte ratio; Pre-IVIG, before intravenous immunoglobulin treatment; Post-IVIG, after intravenous immunoglobulin treatment; FC, fractional changes; IVIG, intravenous immunoglobulin.

To further explore the relationships between clinical indicators and IVIG resistance in KD, the aforementioned predictive variables were analyzed using RCS analysis. Notably, all indicators exhibited significant nonlinear associations with IVIG resistance risk (P < 0.001) (**Figure 3**, **Supplementary Figure 1**). **Figures 3A–C** shows the nonlinear RCS curve between FC variables and IVIG resistance risk. Among these, the OR value of NE count (FC) (**Figure 3B**) is greater than 1 at values greater than -0.69, indicating that insufficient decrease or even further increase in neutrophil count was significantly associated with a higher risk of IVIG resistance. Similarly, WBC (FC) (**Figure 3C**) showed a sharp increase in OR when value >-0.373. NE% (FC) (**Figure 3A**) also showed a significant nonlinear correlation. When NE% (FC)>-0.479, it indicates that the correlation between insufficient decrease or continued increase in NE% and IVIG resistance is more significant. Post-treatment variables also exhibited notable nonlinear relationships with IVIG resistance (**Figures 3D, E**). Hb (post-IVIG) showed an inverse relationship, with resistance risk significantly increasing when Hb levels dropped below 111.16 g/L, suggesting it as a potential marker for IVIG resistance (**Figure 3D**). LMR (post-IVIG) demonstrated that OR increased gradually as LMR exceeded the threshold of 18.5, with a decelerated rise beyond 150, though the risk continued to increase (**Figure 3E**). **Figure 3F** displays the RCS curve for pre-treatment CLR in predicting IVIG resistance. The curve indicated a significant increase in OR when CLR exceeded 19.106, suggesting that elevated CLR before treatment is associated with IVIG resistance, which aligns with its previous ROC performance. Furthermore, variables were categorized based on their inflection points derived from the RCS analysis. As shown in **Table 4**, multivariate logistic regression revealed significant differences in the risk of IVIG resistance across the subgroups of each variable (P < 0.001).

**Table 4:** Multivariate logistic regression regrouped according to the inflection points of variables in the risk of IVIG resistance.

Inflection point	OR (95% CI)	*P*–value
CLR(Pre-IVIG)		
< 19.11	1	Reference
≥ 19.11	2.11 (1.46, 3.09)	<0.001
NE(FC)		
< -0.48	1	Reference
≥ -0.48	2.39 (1.48, 3.95)	<0.001
NE count(FC)		
< -0.69	1	Reference
≥ -0.69	2.49 (1.30, 4.87)	<0.001
WBC(FC)		
< -0.37	1	Reference
≥ -0.37	2.56 (1.50, 4.51)	<0.001
HB(Post-IVIG)		
< 111.16	1	Reference
≥ 111.16	0.53 (0.35, 0.77)	<0.001
LMR(Post-IVIG)		
< 18.50	1	Reference
≥ 18.50	2.50 (1.70, 3.74)	<0.001

IVIG, Intravenous immunoglobulin; Pre-IVIG, Before intravenous immunoglobulin treatment; Post-IVIG, After intravenous immunoglobulin treatment; CLR, C-reactive protein-to-lymphocyte ratio; NE, Neutrophil percentage; NE count, Neutrophil count; WBC, White blood cell count; HB, Hemoglobin; LMR, Lymphocyte-to-monocyte ratio; FC, fractional changes.

### 3.5 Nomogram construction and validation

**Figure 4:**
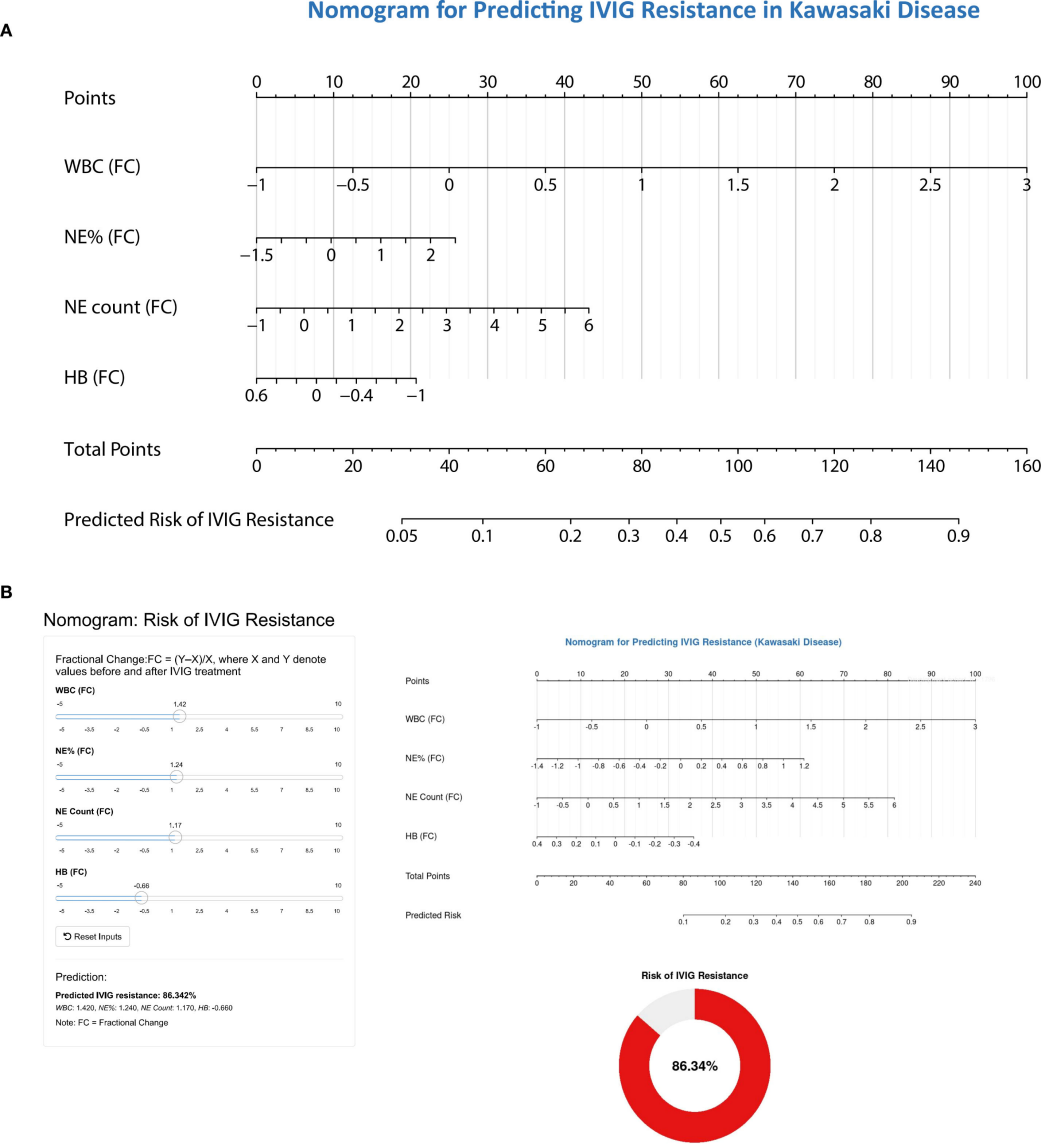
The nomogram for predicting the risk of IVIG resistance in children with Kawasaki disease. **(A)** Composition of predictive nomogram. WBC(FC), fractional change of white blood cell; NE%(FC), fractional change of neutrophil%; NE count(FC), fractional change of neutrophil count; HB(FC), fractional change of hemoglobin concentration. When using a nomograph, the corresponding score is extracted from each risk factor. The total score is obtained by adding the scores of all variables, and the prediction probability is derived based on the position of the total score relative to the baseline reference value. **(B)** Web risk prediction calculator based on predictive nomogram.

**Figure 5:**
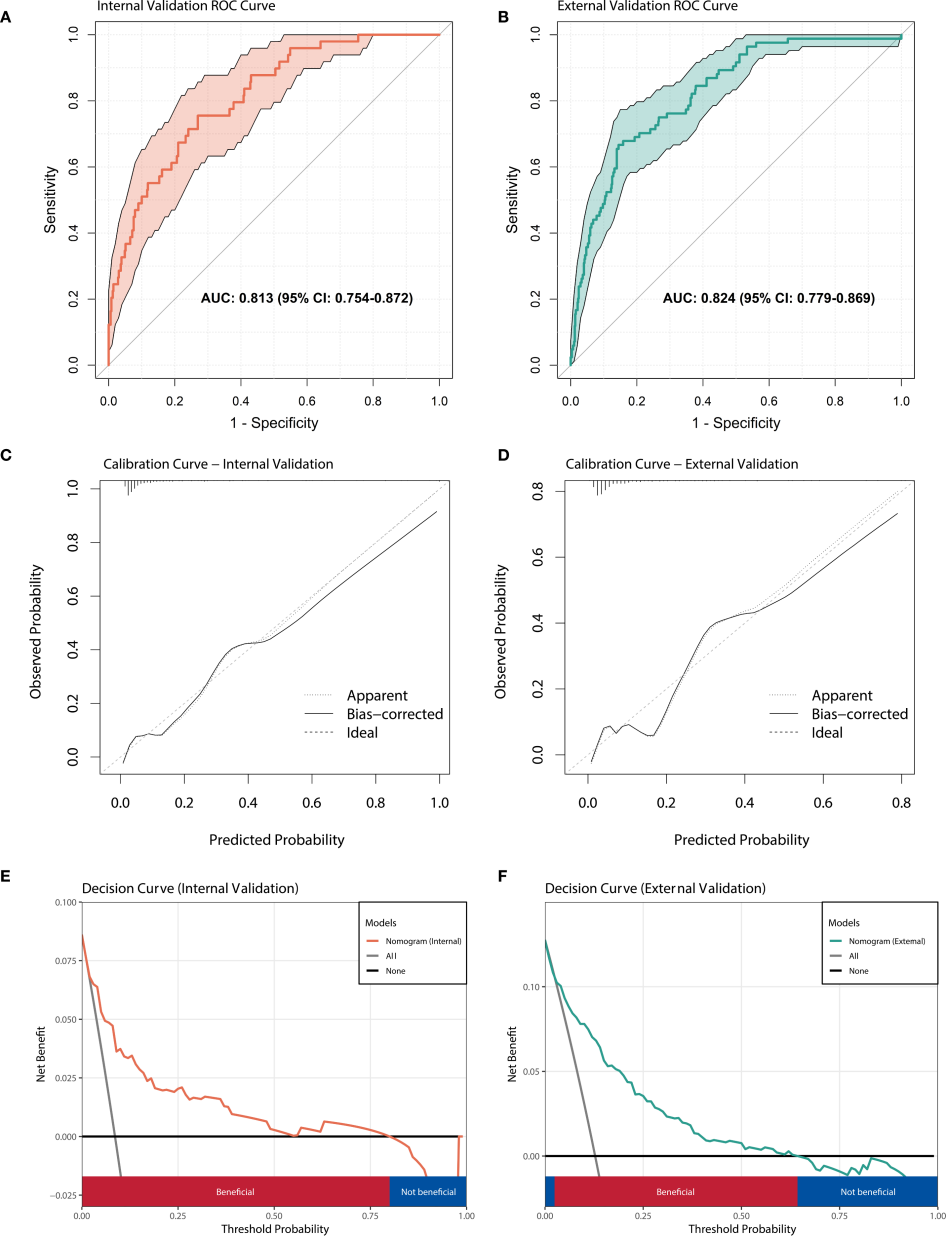
Performance evaluation of nomogram model. **(A)** ROC curve of the prediction model in internal validation set; **(B)** ROC curve of the prediction model in external validation set. **(C)** The calibration curve of prediction model in internal validation set. **(D)** The calibration curve of prediction model in external validation set. **(E)** Decision curve analysis for predicting IVIG resistance in internal validation set. **(F)** Decision curve analysis for predicting IVIG resistance in external validation set.

Based on the results of multivariate logistic regression analysis, a nomogram model containing four variables (WBC(FC), NE%(FC), NE count(FC), HB(FC)) was constructed to predict the risk of IVIG resistance in KD. The predictive ability of each variable is shown in **Figure 4A**, and corresponding scores are assigned. The total score is obtained by adding the scores of all variables, and the prediction probability is derived based on the position of the total score relative to the baseline reference value. Furthermore, in order to facilitate the use of clinicians, this nomogram has been developed into a web-based risk calculator as shown in **Figure 4B** (https://ivig-resistance-prediction-model-fractional-change.shinyapps.io/Kawasaki_nomogram/). The AUC values for the internal and external validation sets were 0.813 (95% CI: 0.754–0.872) and 0.824 (95% CI: 0.779–0.869), respectively (**Figures 5A, B**), indicating good predictive accuracy. This robust performance was further corroborated by the sensitivity and specificity metrics, with the internal set showing a sensitivity of 66.7% and a specificity of 84.6%, while the external set demonstrated a more balanced profile with a sensitivity of 79.8% and a specificity of 75.0%. The calibration curves demonstrated a good agreement between the actual and predicted values of the model (**Figures 5C, D**), suggesting that the model also exhibits good calibration. In addition, the DCA curve shows the significant positive net benefit of the prediction model, indicating that it has good clinical value in predicting IVIG resistance (**Figures 5E, F**).

Besides, we compared our model with previous IVIG resistance scoring systems. **Supplementary Table 2** presents the analysis results of the Egami, Sano, Kobayashi, and Formosa scoring systems in our external validation set. The Egami score system, which exhibits the best performance, has a sensitivity of 50.5%, specificity of 83.2%, and AUC of 0.667. However, compared with these classic prediction models, it can be found that the model in this study has more balanced sensitivity, specificity, and higher AUC values.

## 4 Discussion

The immunopathogenesis and regulatory mechanisms of inflammation in KD remain unclear. Previous studies have suggested that the occurrences of IVIG resistance in KD were associated with the intensity of the inflammatory response (19–21). Similarly, the studies of Cho KH et al. (22) also reported the correlation between fractional changes in some inflammatory indicators and IVIG resistance. However, these fractional changes with observed correlations have not yet been applied to KD-related predictive models. Even its predictive value for IVIG resistance in KD children also has not been evaluated. Therefore, this study primarily investigates the predictive value of fluctuations in inflammatory parameters before and after IVIG treatment for IVIG resistance in children with KD. Our findings underscore the variability and complexity of immune inflammation in KD, emphasizing the importance of dynamic evaluation to enhance the accuracy of clinical outcome predictions. To our knowledge, this is the first study to demonstrate that fractional changes in leukocyte counts, particularly the NE count, before and after IVIG treatment exhibit strong predictive potential for IVIG resistance.

Neutrophils contribute to host defense mechanisms through processes such as chemotaxis, activation, degranulation, and oxidative burst (23). However, in inflammatory diseases, neutrophil functions can become excessively activated or dysregulated, leading to tissue damage and pathological inflammation. Recent research also highlights the significant role of neutrophil-specific inflammatory subsets and neutrophil extracellular traps (NETs) in KD progression (24). Consistent with prior studies, our results also show that WBC and neutrophils play an important role in the pathogenesis of Kawasaki disease, particularly their predictive value for IVIG resistance (25, 26). A recent meta-analysis of 114,000 children with KD across 14 studies confirmed the significant correlation between neutrophils and IVIG resistance prediction (27).

In contrast to these studies, our findings emphasize the remarkable predictive capacity of fractional changes in WBC, particularly in neutrophil counts, before and after treatment for IVIG resistance. For instance, Guo et al. reported the AUC value of WBC before treatment in predicting IVIG resistance was only 0.59 (28). In contrast, the AUC value for WBC (FC) reached 0.77 in our study, similar to the predictive ability of the model constructed in their study (AUC = 0.758). This significant difference may arise from the fact that prior studies primarily relied on single time-point measurements of clinical variables, which may not adequately capture the dynamic nature of KD. In our study, WBC (FC) is derived from two separate measurements, reflecting the disease characteristics at two distinct time points. Thus, when constructing prediction models for KD in future studies, fractional change should be incorporated into clinical variables to improve the predictive accuracy for IVIG resistance.

CLR reflects systemic inflammation, with elevated CRP indicating an inflammatory state, while lymphocyte suppression suggests immune dysfunction (29). CLR has previously been identified as a prognostic biomarker in acute pancreatitis and as a diagnostic and prognostic tool for dilated cardiomyopathy (30, 31). Likewise, LMR is the ratio of lymphocytes to monocytes, where a decrease in lymphocytes typically indicates an immunosuppressive state, while an increase in monocytes reflects persistent inflammatory activation (32). Over recent years, LMR has demonstrated potential as a effective biomarker for the diagnosis and prognosis of several malignancies and infectious diseases (32, 33). Research by Wu et al. and Xu et al. have demonstrated CLR and LMR as reliable biomarkers for differentiating KD from other febrile illnesses (34, 35). Building on this foundation, the current study evaluates their predictive value in KD. Multivariate logistic regression identified pre-treatment CLR and post-treatment LMR as independent predictors of IVIG resistance, with ROC analysis confirming their predictive potential. Although the underlying immunological mechanisms remain unclear, CLR and LMR, as cost-effective and easily accessible biomarkers, warrant further investigation in KD.

This study is the first to identify pre-treatment CLR and post-treatment LMR as predictors of IVIG resistance in KD. However, their predictive performance was not superior to WBC (FC) and NE count (FC). Numerous prior studies have explored molecular and immunological markers for predicting IVIG resistance in KD, yet their predictive performance has often been inconsistent or unsatisfactory, limiting their clinical applicability (12, 13, 28). In addition to genetic and environmental factors, the reliance on cross-sectional indicators measured at a single time point remains a significant limitation. Given the dynamic nature of inflammatory indicators in KD, a single measurement may fail to accurately reflect the child’s immune status or capture sufficient clinical features to predict IVIG resistance. This limitation was also noted in Pang et al.’s study (36), but their conclusion regarding the predictive value of fractional changes in parameters differed from ours. The discrepancies could be attributed to their smaller sample size of 153 cases, which may have reduced statistical power and increased the influence of random variability, potentially leading to overestimation or underestimation of the true predictive performance (AUC). Moreover, consistent with previous studies, we also demonstrated a significant nonlinear relationship between clinical parameters and risk of IVIG resistance (37, 38). Such non-linear associations further complicate prediction efforts based on single-time point measurements. As illustrated in **Figure 3B**, even parameters with robust predictive capabilities may yield ineffective or opposite results before reaching a specific threshold or inflection point, thus partly explaining the inconsistent predictive performance of various biomarkers or models across children with KD.

The non - linear trends and inflection points observed in the NLR and hemoglobin fractional change following IVIG administration have significant implications for clinical decision - making and their potential inclusion in risk stratification frameworks.

Regarding the non - linear trend of NLR, an initial increase followed by a decrease or vice - versa may not be a simple linear response to IVIG treatment. For instance, an early rise in NLR could indicate an initial pro - inflammatory state triggered by the immune modulation induced by IVIG. This might prompt clinicians to closely monitor the patient for signs of worsening inflammation, such as increased fever or organ dysfunction. If the NLR then shows a non - linear decline, it could suggest a successful anti - inflammatory effect of IVIG over time.

The inflection points in the hemoglobin fractional change also carry clinical weight. A sharp decline in hemoglobin (FC) could be a critical event associated with IVIG resistance or hemolysis, which can be an adverse reaction to IVIG in some cases. Clinicians should be alerted to this inflection point and immediately investigate the cause. Furthermore, these non-linear trends and inflection points can be incorporated into existing or new frameworks in terms of risk stratification. For instance, patients with a more pronounced non-linear increase in NLR or a very sharp decline in hemoglobin FC could be classified as high-risk. This high-risk classification would then guide more intensive monitoring, such as more frequent laboratory tests and clinical assessments, and potentially more aggressive treatment strategies. By integrating these dynamic biomarkers into risk stratification, clinicians can better tailor their management approaches to individual patients, optimizing treatment outcomes and resource utilization. Therefore, it is essential to develop prediction models with appropriate thresholds and dynamic risk assessment in the future. Establishing appropriate thresholds can enhance the predictive performance of model parameters. Additionally, dynamic evaluation at multiple time points not only offers more precise guidance for clinicians in crafting individualized treatment strategies for patients with KD but also enables real-time monitoring of patients’ inflammatory status, thereby optimizing the timing of interventions for IVIG-resistant patients. For clinicians, more attention should be paid to those children with risk factors of IVIG resistance, and the risk of IVIG resistance should be evaluated again after treatment. Notably, fractional changes in predictors before and after treatment may offer superior predictive value for IVIG resistance.

This study also has several limitations. First, as a retrospective study, the findings may be subject to selection bias. Second, although multivariate logistic regression models were used to control confounding factors, our results may still be influenced by unmeasured or unknown environmental variables. Third, it’s important to note that our study is exclusively based on the Chinese population. Given the well - documented ethnic and geographical differences in disease prevalence, manifestations, and genetic susceptibilities, there is significant uncertainty regarding the applicability of our findings in non - Asian populations. Different ethnic groups may have distinct genetic backgrounds, lifestyles, and environmental exposures, all of which can potentially impact the development, progression, and characteristics of the disease under investigation. As a result, the conclusions drawn from our Chinese - centric study may not be directly generalizable to other racial or ethnic groups, and further research in diverse populations is warranted to validate and extend our results. Fourth, genetic variability among individuals is unavoidable, and the values of the same blood parameters may fluctuate differently in different children. Future studies could consider carrying out prospective cohort studies and identify the potential multiple subtypes of children with Kawasaki disease through trajectory modeling. In this study, we used Z ≥ 2 as the cut-off value to define CAL. However, the 2017 AHA guidelines defined aneurysms (CAA) as having a Z score of ≥fore and further classified them into small, medium, and large categories based on size. In this study, by combining Z ≥2.0 and Znd.0 into the category of CAL, we must admit that the reported incidence of CAL in our study may have been overestimated due to the inclusion of minor and often transient expansions.

## 5 Conclusion

In conclusion, this study underscores the importance of dynamic evaluation of inflammatory biomarkers in predicting IVIG resistance. Specifically, fractional changes in WBC and neutrophil counts before and after treatment were identified as independent risk factors and effective predictors. Furthermore, the observed nonlinear relationship between biomarkers and IVIG resistance underscores the necessity of establishing appropriate thresholds in evaluations. In the future, it is essential to develop a prediction model with accurate thresholds and dynamic risk assessments to improve the prediction ability of IVIG resistance.

## Data Availability

The raw data supporting the conclusions of this article will be made available by the authors, without undue reservation.

## Supplementary material

Supplementary Figure 1 RCS analysis of additional predictive variables for IVIG resistance. **(A)** Correlation of Hb (Pre-IVIG) with IVIG resistance. **(B)** Correlation of CLR (Post-IVIG) with IVIG resistance. **(C)** Correlation of NLR (Post-IVIG) with IVIG resistance. **(D)** Correlation of Hb (FC) with IVIG resistance. Hb, hemoglobin; CLR, C-reactive protein-to-lymphocyte ratio; NLR, neutrophil-to-lymphocyte ratio; Pre-IVIG, before intravenous immunoglobulin treatment; Post-IVIG, after intravenous immunoglobulin treatment; FC, fractional changes; IVIG, intravenous immunoglobulin.
