# Supplementary material for: Value of dynamic changes in inflammatory biomarkers for predicting intravenous immunoglobulin resistance in children with Kawasaki disease
Source: Front Immunol. 2025 Sep 29;16:1632578. doi: 10.3389/fimmu.2025.1632578 (PMC12515928; doi:10.3389/fimmu.2025.1632578)
Supplement: Supplementary Figure 1 — RCS analysis of additional predictive variables for IVIG resistance. (A) Correlation of Hb (Pre-IVIG) with IVIG resistance. (B) Correlation of CLR (Post-IVIG) with IVIG resistance. (C) Correlation of NLR (Post-IVIG) with IVIG resistance. (D) Correlation of Hb (FC) with IVIG resistance. Hb, hemoglobin; CLR, C-reactive protein-to-lymphocyte ratio; NLR, neutrophil-to-lymphocyte ratio; Pre-IVIG, before intravenous immunoglobulin treatment; Post-IVIG, after intravenous immunoglobulin treatment; FC, fractional changes; IVIG, intravenous immunoglobulin. [file DataSheet1.docx]

Supplementary Material

**Supplementary Table 1 Univariate and multivariate logistic analysis for predictors of IVIG resistance before and after IVIG**

| **Characteristics** | **Univariable** | |  | **Multivariable** |  |  | Adjust# |  |  | **VIF** |
| --- | --- | --- | --- | --- | --- | --- | --- | --- | --- | --- |
|  | **odds ratio (95%CI)** | ***P*–value** |  | **odds ratio (95%CI)** | ***P*–value** |  | **odds ratio (95%CI) *P*–value** | |  |  |
| CLR ( Pre-IVIG ) | 1.008(1.005-1.011) | ＜0.001 |  | 1.005 (1.001-1.009) | 0.011 |  | 1.005 (1.001-1.009) | 0.009 |  | 2.064 |
| PLR ( Pre-IVIG ) | 1.003(1.002-1.005) | ＜0.001 |  | 1.001 (0.997-1.003) | 0.624 |  | 1.000 (0.997-1.003) | 0.653 |  | 2.344 |
| PLT(x109/L) ( Pre-IVIG ) | 0.997(0.996-0.999) | 0.001 |  | 0.998 (0.996-1.000) | 0.062 |  | 0.998 (0.996-1.000) | 0.065 |  | 1.358 |
| LY count, (x109/L)( Pre-IVIG) | 0.811(0.732-0.892) | ＜0.001 |  | 0.959 (0.825-1.008) | 0.539 |  | 1.005 (0.972-1.024) | 0.637 |  | 1.03 |
| LY, % ( Pre-IVIG) | 0.973(0.959-0.987) | ＜0.001 |  | 1.005 (0.972-1.023) | 0.667 |  | 0.960 (0.826-1.008) | 0.544 |  | 4.456 |
| NE, % ( Pre-IVIG) | 1.026(1.014-1.038) | ＜0.001 |  | 1.008 (0.979-1.028) | 0.505 |  | 1.008 (0.980-1.029) | 0.476 |  | 4.802 |
| HB (g/L) ( Pre-IVIG) | 0.983(0.968-0.997) | 0.02 |  | 0.978 (0.962-0.992) | 0.003 |  | 0.977 (0.962-0.992) | 0.003 |  | 1.034 |
| EO, % ( Pre-IVIG) | 0.577(0.328-0.924) | 0.038 |  | 0.776 (0.446-1.205) | 0.32 |  | 0.772 (0.444-1.199) | 0.312 |  | 1.127 |
| CLR (Post-IVIG) | 1.012(1.001-1.025) | 0.046 |  | 1.035 (0.997-1.072) | 0.049 |  | 1.036 (0.998-1.073) | 0.042 |  | 1.245 |
| LMR (Post-IVIG) | 1.004(1.003-1.006) | ＜0.001 |  | 1.003 (1.002-1.005) | ＜0.001 |  | 1.003 (1.002-1.005) | ＜0.001 |  | 1.068 |
| SII (Post-IVIG) | 1.001(1-1.001) | ＜0.001 |  | 1.001 (0.999-1.002) | 0.116 |  | 1.001 (0.999-1.002) | 0.122 |  | 1.472 |
| PLR (Post-IVIG) | 0.994(0.99-0.998) | 0.003 |  | 0.995 (0.985-1.001) | 0.289 |  | 0.995 (0.985-1.001) | 0.302 |  | 1.379 |
| NLR (Post-IVIG) | 1.282(1.162-1.418) | ＜0.001 |  | 0.360 (0.167-0.712) | 0.006 |  | 0.357 (0.166-0.708) | 0.005 |  | 1.024 |
| PLT(x109/L) (Post-IVIG) | 1.001(1-1.002) | 0.017 |  | 0.998 (0.996-1.000) | 0.3 |  | 0.998 (0.996-1.001) | 0.3 |  | 1.346 |
| HCT (Post-IVIG) | 0(0-0.001) | ＜0.001 |  | 5.244 (0.000-3896.) | 0.669 |  | 4.105 (0.000-3334.) | 0.719 |  | 1.134 |
| HB (g/L) (Post-IVIG) | 0.962(0.948-0.975) | ＜0.001 |  | 0.970 (0.948-0.996) | 0.01 |  | 0.970 (0.947-0.995) | 0.01 |  | 1.081 |
| WBC(FC) | 4.21(3.175-5.641) | ＜0.001 |  | 4.2(2.836-6.318) | ＜0.001 |  | 4.201 (2.835-6.325) | ＜0.001 |  | 1.704 |
| HB(FC) | 0.062(0.012-0.312) | 0.001 |  | 0.066(0.012-0.372) | 0.002 |  | 0.065 (0.012-0.371) | 0.002 |  | 1 |
| CRP(FC) | 1.477(1.166-1.952) | 0.002 |  | 1.214(1.018-1.641) | 0.157 |  | 1.213 (1.017-1.641) | 0.159 |  | 1.039 |
| NE%(FC) | 2.065(1.436-3.038) | ＜0.001 |  | 2.021(1.119-3.624) | 0.014 |  | 2.021 (1.119-3.623) | 0.014 |  | 2.859 |
| NE count(FC) | 1.722(1.392-2.143) | ＜0.001 |  | 0.758(0.562-0.981) | 0.04 |  | 0.758 (0.562-0.981) | 0.04 |  | 1.946 |
| PLR(FC) | 0.542(0.391-0.73) | ＜0.001 |  | 0.767(0.565-0.958) | 0.067 |  | 0.767 (0.564-0.958) | 0.067 |  | 1.001 |
| LMR(FC) | 1.017(1.007-1.029) | 0.003 |  | 1.01(1-1.02) | 0.053 |  | 1.009 (0.999-1.020) | 0.054 |  | 1.019 |
| Adjust#: Adjusted odds ratio calculated using multivariable logistic regression analysis, adjusting for age, gender, and duration of fever.  Abbreviations: IVIG, Intravenous immunoglobulin; CLR, C-reactive protein-to-lymphocyte ratio; PLR, Platelet-to-lymphocyte ratio; PLT, Platelet count; LY count, Lymphocyte count; LY, Lymphocyte percentage; NE, Neutrophil percentage; HB, Hemoglobin; EO, Eosinophil percentage; LMR, Lymphocyte-to-monocyte ratio; SII, Systemic immune-inflammation index; NLR, Neutrophil-to-lymphocyte ratio; WBC, White blood cell count; HCT, Hematocrit; FC, Fold change; CRP, C-reactive protein; VIF: Variance inflation factor; Pre-IVIG, Before intravenous immunoglobulin treatment; Post-IVIG, After intravenous immunoglobulin treatment; FC, Fractional changes. | | | | | | | | | | |

| **Supplementary Table 2 Comparison between the our model and other IVIG resistance KD prediction models.** | | | | | | | |
| --- | --- | --- | --- | --- | --- | --- | --- |
| **Models** | **Sensitivity(%)** | **Specificity(%)** | **AUC** | **95% CI Lower** | **95% CI Upper** | **PPV** | **NPV** |
| **Egami score** | 50.5% | 83.2% | 0.667 | 0.611 | 0.723 | 0.455 | 0.858 |
| **Sano score** | 28.2% | 97.2% | 0.619 | 0.562 | 0.676 | 0.273 | 0.974 |
| **Kobayashi score** | 47.5% | 87.1% | 0.649 | 0.592 | 0.706 | 0.315 | 0.930 |
| **Formosa** | 48.1% | 70.6% | 0.592 | 0.534 | 0.650 | 0.274 | 0.855 |
| Abbreviations: AUC,area under the curve;PPV, positive predictive value; NPV, negative predictive value. | | | | | | | |


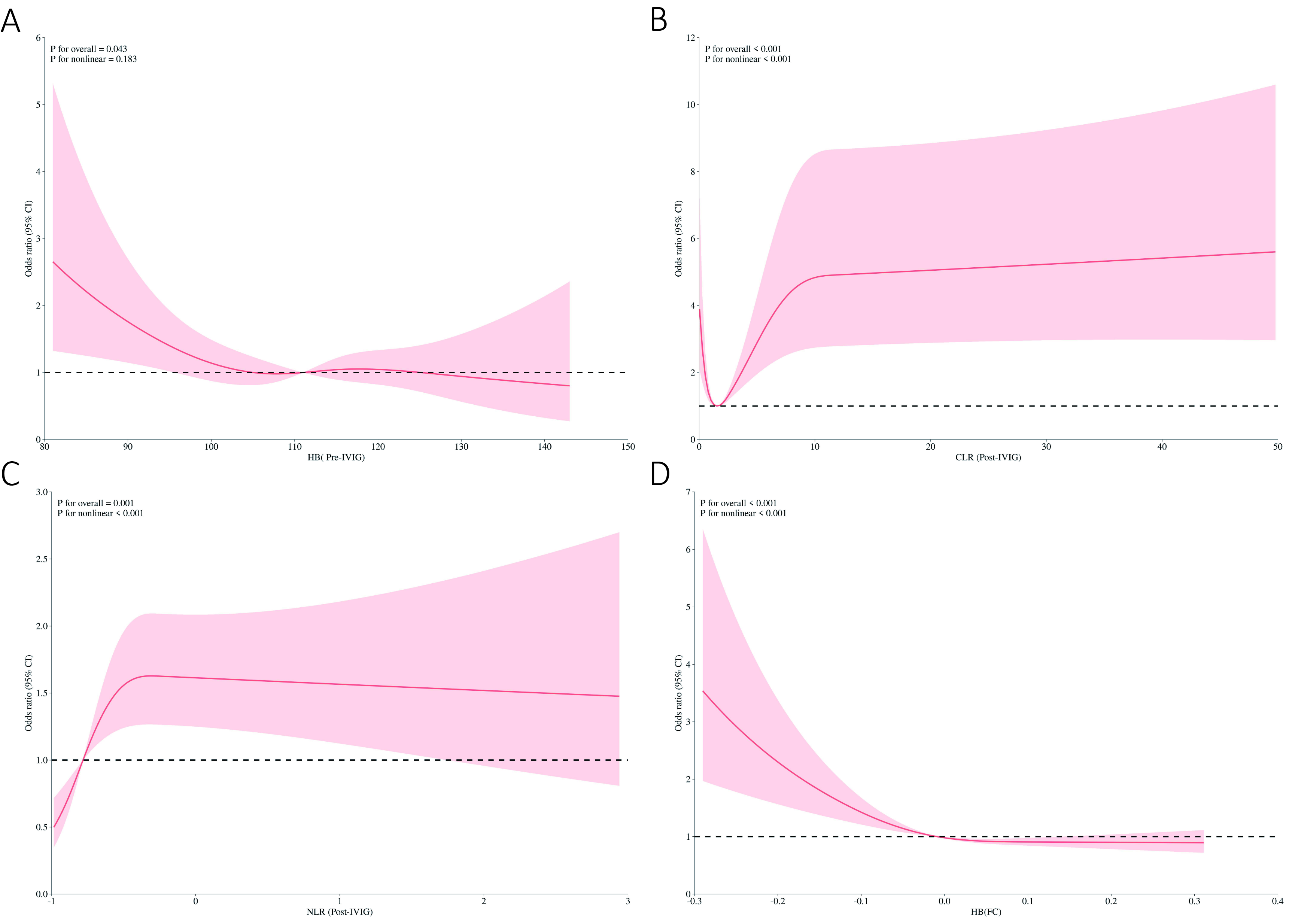


**Supplementary Figure 1**. RCS analysis of other predictive variables for IVIG resistance.(A) Correlation of HB (Pre-IVIG) with IVIG resistance.(B) Correlation of CLR(Post-IVIG) with IVIG resistance.(C) Correlation of NLR(Post-IVIG) with IVIG resistance.(D) Correlation of HB(FC) with IVIG resistance.Hb, Hemoglobin;CLR,C-reactive protein-to-Lymphocyte Ratio;NLR, Neutrophil-to-Lymphocyte Ratio;Pre-IVIG, Before intravenous immunoglobulin treatment; Post-IVIG, After intravenous immunoglobulin treatment;FC, fractional changes;IVIG, intravenous immunoglobulin.
